# Report on the Progress of Pathological Anatomy*This report embraces the years 1850–59. As it was prepared several months ago, the latter part of 1858 is not included.

**Published:** 1859-03

**Authors:** J. Da Costa


### American Progress of Medical Science.

### REPORT ON THE PROGRESS OF PATHOLOGICAL ANATOMY.*

By J. Da Costa, M.D.

The subject of Pathological Anatomy, from its intimate connection with diagnosis, is one which has engaged the attention of the master-minds of the profession ever since medicine has been cultivated as a science. Its importance in this age of research and of sifting of theories has been especially felt; and it would not be a difficult matter to prove that its present cultivation has been one of the main causes in bringing about that accuracy of diagnosis, and the more correct appreciation of the value of remedies, which is the pride of modern medicine. In most of the cities of Europe its value is testified to by the valuable contributions annually made to it, and by the institution of chairs from which it is taught as a separate branch. In this country such is not, with few exceptions, the case; but that the medical mind is alive to the importance of the study, is shown by the establishment, in some of our larger cities, of Pathological Societies, which have already given evidence of so much vitality, that a still brighter future may be safely predicted for them. The establishment of these societies is undoubtedly a means of increasing and diffusing pathological knowledge: yet it is very desirable that those whose opportunities permit of it, should contribute connected observations, and not merely isolated facts, whose value for the positive advancement of the science is, after all, but fractional, since, with rare exceptions, they furnish but little toward the elucidation of those laws governing pathological processes, which are so important in understanding, and in endeavoring to counteract the natural tendencies of disease.

We may be pardoned for this digression at the commencement of what is intended to be simply an analytical record of the progress of Morbid Anatomy during the last eight or nine years in this country. But we have been struck with the circumstance alluded to in examining the periodicals for our report. While we have met with many most valuable isolated observations scattered over the various journals, we have found but few papers discussing in a general manner points connected with Pathology or Pathological Anatomy. Sometimes even the descriptions of the post-mortem appearances appended to cases whose interest during life was very great, are so hurried as to throw no light whatever on the real nature of the disease. Having thus indicated the material we have had to select from, it will be seen that two methods of writing this record presented themselves—either to confine ourselves strictly to an analysis of the papers on pathological subjects, or of exclusively pathologico-anatomical observations

---

* This report embraces the years 1850–59. As it was prepared several months ago, the latter part of 1858 is not included.

which have appeared, or else, in addition, to classify and connect those cases of interest in which the post-mortem appearances are described.

After some reflection we chose the latter, believing that, although far more laborious, it would be of more value, and accomplish more satisfactorily the object of the report. It is evident that we had to content ourselves frequently with mere references, and that only to the more interesting cases, for it is equally evident that had we attempted to transfer, or even cite every case in which the post-mortem appearances are given, all the pages of this journal would hardly have sufficed. The rule we have endeavored to follow has been, to place at the head of each section the title and references to those papers which treat of the subject in a comprehensive way; then briefly to indicate their contents; while the morbid appearances in isolated cases are usually referred to in the body of the report. It is of course not possible to affirm, that where we have had to examine so many publications, nothing valuable has escaped us; doubtlessly that will be found in some instances to have been the case; but we trust that we have been successful in collecting together the main facts of interest, and that in this respect our record, while it neither claims to be exhausting, nor even fully analytical, may at least serve as a classified bibliographical index to the more important of the subjects discussed. Cases, whose interest lay more in their history, or in their elucidating some special point of treatment or diagnosis in surgery, obstetrics, or the practice of medicine, have been briefly or not at all touched upon. Indeed, the very word *case*, that we have been so frequently obliged to use, suggests to us that the peculiar character of the materials used have given to this report, in many respects, less of a strictly pathological, and somewhat more of a clinical bearing than records of this nature are intended to possess.

Not to drag out this introduction to a tedious length, we would merely add, that the classification was more prompted by convenience than intended to be scientifically accurate, and that the task we have set before us has been that of an analyser, and not that of a critic.

In reviewing the different works which have appeared in this country on Pathological Anatomy, we have, in addition to translations and reprints of standard foreign works, such as of Hasse, Baillie, Craigie, Jones, Sieveking, and Rokitansky, the well-known volumes of Horner and Gross. Nor must we omit to mention here the descriptive catalogue of the museum of the Boston Society for Medical Improvement, (Boston, 1847,) arranged by Dr. Jackson, which contains a large number of valuable facts, with the history of many of the cases attached, and is thus one of the good fruits which this active and valued society has borne. Most of the other contributions have appeared in the pages of medical journals; and we trust it may not be considered presumptuous if we express a hope that another society, whose zeal and value is appreciated everywhere, the Pathological Society of New York, would record in a permanent form, by collecting in a volume, those interesting observations and facts which the opportunities of a large city, and the zeal of the members, have brought together.

### GENERAL PATHOLOGICAL ANATOMY.

So few papers have appeared that would legitimately come within the scope of this part of our report, that it would be useless to attempt to classify them. We shall content ourselves, therefore, with simply placing such together as treat more or less on similar subjects, or such observations whose interest lies in their contributing to the study of some point of general morbid change.

### MONSTROSITIES—MALFORMATIONS.

*S. S. Purple.*—"A Literary, Historical, and Practical Sketch of Acrania, or Pseudo-encephalous Monsters;" New York Journal of Medicine, vol. iv., 1850.

*George C. Blackman.*—"On Hermaphroditism;" Am. Journ. of Med. Sciences, July, 1853.

*Charles D. Meigs and G. W. Boerstler.*—"Case of a Monstrous Birth, with Remarks;" Am. Journ. of Med. Sciences, July, 1855.

*A. Jacobi.*—"On Irregular Development of the Infantile Cranium;" New York Journal of Medicine, January, 1858.

The subject of monstrosities is one which, in its descriptive parts, has received considerable attention; in its more general and philosophical bearings, but little. Dr. Purple, in the paper quoted, discusses the subject of brainless monsters in its pathological and medico-legal relations, analyzes some of the reported cases, and adds the description of a fœtus born of a healthy mother after a labor of only thirty minutes, in which the frontal and squamous portion of the temporal bones, together with the superior portion of the occipital and the contents of the cranium, seemed to be wanting down to a point level with the base of the cranium; this was covered with a thin membrane connected to the skin. Over the site of the superior portion of the medulla oblongata, a vascular tumor was seen; pressure upon this tumor gave rise to convulsive action of the whole system. The child lived two days and a half.

Dr. John C. Dalton, Jr.* has detailed an interesting case of malformation of the cranium, encephalon, and spinal cord. Every part of the fœtus, externally, was perfect, excepting the face and cranium. The coverings of the brain partly resembled skin, and partly serous membrane.

An anencephalous fœtus was dissected by Dr. Chas. E. Isaacs.† Not the slightest trace of a head could be found; its place was occupied by a quantity of cellular tissue, covered by very smooth integument. There was no heart, and the place of the lungs was supplied by small masses of cellular tissue. The spleen also was absent. Another specimen of the kind is described by the same author,‡ and further interesting cases of monstrosities reported by Dr. J. W. B. Garrett,§ and by Drs. G. W. Garland|| and W. F. Atlee.¶ In Dr. Atlee's

---

* American Journal of Medical Science, April, 1850.

† See New York Journal of Medicine, vol. iv. p. 331.

‡ Ibid., vol. x. p. 205; 1853.

§ Western Journ. of Med., July, 1850.

|| Ibid., vol. vii. p. 212; 1851.

¶ Am. Journ. of Med. Science, April, 1858.

valuable case, the monster belonged to the genus of peracephalus, (St. Hilaire,) having no head or upper extremity.

Dr. Charles D. Meigs* describes a double-headed anencephalous fœtus, with two well-formed arms and legs, and a single trunk; and in another communication,† a specimen presented to him by Dr. Boerstler, with two heads, four arms, two chests down to the ensiform cartilages, but from the junction downward only one body, its anterior surface, from side to side, broader than in an ordinary child; two perfect spinal columns; two pelves united, the left one belonging to the larger side, encroaching upon, and lessening that of the right side. The sex was female; one vulva and one anus; the opening of the latter not larger than an ordinary rye-straw. In addition to the two legs, there arose from the dorsum of the two ossa ilia, a leg which ran up between the two spines and the two inner shoulders, and which terminated in a right and left foot joined at the heel. This was, in truth, a double leg, having two femurs, two tibias, and two fibulas. Not the least remarkable circumstance about this curious monstrosity was that the children lived for upwards of a month.

In a fœtus which Dr. G. F. Fisher‡ has described, two heads, both hydrocephalic, were attached, each by a distinct neck, to a body which was single. The thoracic cavity contained one large heart. A monster in some respects very much like that of Prof. Meigs's, is described by Dr. Jas. B. Dungan.§

Dr. J. B. S. Jackson|| has given an instance of malformation in an adult subject, otherwise well formed, consisting apparently of a fusion of the two upper extremities.

One of Dr. Blackman's cases of malformation of the sexual apparatus is truly remarkable. In the body of a person about twenty-six years of age there was a penis, an empty scrotum, a prostate gland, a vagina, an os tincæ, a uterus, a bladder, right and left Fallopian tubes, right and left testicles, right and left ovaries, a rectum, a right and a left vas deferens. The vagina opened into the neck of the bladder, and thus communicated with the urethra; its inner surface was reddened, and its cavity contained menstrual blood. The Fallopian tubes were pervious. Dr. Burnett,¶ who also examined the organs, states the vagina to have been of the usual size, but contracted at its lower part, into a small tube, which passed into the urethra between the lobes of the prostate glands. Dr. J. B. S. Jackson** investigated the same specimen, and found some thickening of the tissues where the ovaries should be, but it was ill defined and slight. Upon one side an incision was made into this questionable part, but nothing like a Graafian vesicle was seen, merely a loose fibro-cellular tissue; a yellow body was exposed, which was thought at first to be a corpus luteum, but proved to be a lobule of fat.

---

* Am. Journ. of Med. Science, Jan. 1857 † Ibid., July, 1855.

‡ American Medical Monthly, October, 1857, p. 229.

§ New Orleans Medical News, May, 1857.

|| Society for Medical Improvement; Am. Journ. of Med. Science, January, 1853, p. 91.

¶ Ibid., October, 1853, p. 368.

** Am. Journ. of Med. Science, October, 1854, p. 369.

While speaking of the malformations of special organs, we may allude to Dr. Jacobi's researches, who has brought together valuable information on the irregular development of the infantile cranium.

TEXTURAL CHANGES—NEW FORMATIONS—TUMORS.

*W. J. Burnett.*—"Tissue, and its Retrograde Metamorphosis;" Am. Journ. of Med. Science, July, 1851.

*J. W. C. Ely.*—"On Fatty Degeneration;" Am. Journ. of Med. Science, January, 1854.

*W. J. Burnett.*—"The Microscopical and Histological Relations of Enchondroma;" Am. Journ. of Med. Science, April, 1852.

*Francis Donaldson.*—"The Practical Application of the Microscope to the Diagnosis of Cancer;" Am. Journ. of Med. Science, January, 1853.

*J. H. Brinton.*—"Microscopical Observations of Tumors;" Medical Examiner, November, 1851.

*J. Da Costa.*—"Remarks on Epithelial Tumors and Cutaneous Cancers;" Medical Examiner, April and May, 1852.

*J. J. Woodward.*—"Minute Anatomy of Cysto-carcinoma;" Am. Journ. of Med. Science, July, 1858, and sequel to one of the cases. "Histological Observations on a Secondary Cancer of the Pleura;" October, 1858.

The paper of Dr. Burnett on tissue, and its retrograde metamorphosis, is one in which especially the fatty degeneration to which organs are liable, is described. "Constant practice in microscopical pathology," says the author, "is all the while convincing me of the frequency of this change in tissues, and that it may serve as the basis of many accidents in medicine," an opinion which the increasing research of every year has more and more verified. In all the instances of fatty degeneration of muscle met with, the cause could be traced either to a total disuse of the part, or to a previously existing inflammation, leaving the tissue with a poor and imperfect nutrition, or to a starvation of the part from local arterial obstruction. The causes and the appearances of parts fattily degenerated, are further considered in the summary of the subject which Dr. Ely has published.

Dr. Burnett reviews the appearances of the tumors denominated enchondromatous. He regards as doubtful many of the cases which have been recorded as cartilaginous formations, such as the growths in the tunics of the testicle, by Cooper, and the alleged cartilaginous tumors found in the peritoneal cavity. Several of these, from his own examinations, he states to have been of a fibrous nature. He gives the microscopic appearances of two cases of true enchondroma of the hand, similar in many respects to the classical description of Mueller; both of them contained cartilage corpuscles lying in granular stroma.

The subject of *cancer*, one of favorite investigation with pathologists of all ages, has had at no time more labor bestowed on it than since the application of the microscope to pathological researches. This instrument has shown us a certain group of elements constantly occurring in cancer, and which have, therefore, been regarded by many as characteristic. Especially has this been the case with the so-called cancer-cells. Dr. Donaldson, after insisting strongly upon the value of the microscope in the diagnosis of different growths, sides with those French observers who have described particular elements in cancer, and accounts for the difficulty which some have had of detecting them with accuracy, by the fact that they employed too feeble a magnifying power. He meets the objection of those who state that the microscope can be of no service in the living subject, by citing several cases in which it revealed evidences of cancer in small portions removed prior to the operation. He then proceeds to analyze the elements of cancer, speaking of the cancer nucleus as inclosed in a cell, or as floating free by itself; the polygonal, or more or less spherical and ovoid cells, the caudated cell, the fusiform cell, the concentric cell, the compound or mother cell, and the agglomerated nuclei connected by amorphous tissue. The nucleoli found in the cancer nuclei, are mentioned as of a yellowish tinge, with a brilliant centre and dark borders, refracting light like the fat vesicles. The peculiar brilliancy of the centres of these nucleoli, Dr. Donaldson regards as characteristic. The author then discusses, and illustrates by comparative drawings, the corpuscles which have been mistaken for cancer, such as the fibro-plastic cells, pus cells, and cartilage elements, which have been compared by Bennett to cancer cells. He does not understand how these latter can be taken for cancer, nor is he inclined to adopt Schwann's theory of cell-generation in cancer. On the whole, the essay supports the opinions of Lebert and Robin, and is corroborative of a special element characteristic of cancer.

Dr. Woodward describes the appearances of three cases of cysto-carcinoma, representing different stages in the development of this affection. In the first the tumor consisted of a fibrous cyst or sac, almost entirely filled with a clear, liquid, of a ruddy straw-color, which coagulated spontaneously on standing. In the upper part of the cyst was a soft pultaceous mass, composed of granules and nuclei, imbedded in a transparent matrix. In the second case, soft pultaceous masses adhered everywhere to the internal surface of the cyst, and yielded the same appearance under the microscope. The cyst wall was composed of white fibrous, mingled with yellow elastic tissue, with embryonic elements in such abundance, as to prove clearly that it was to a great extent a new formation. In a third case the whole cyst was filled with a soft, brain-like matter. These cases differ from ordinary cancer formations by the presence of a sac, and of an unusual quantity of blastema. In the first case a secondary cancer of the pleura was developed. Everywhere adherent to that membrane were found soft pultaceous masses, exactly in their minute appearance like those observed projecting into the cyst of the primary tumor. In the quantity of blastema this secondary formation was also allied to the primary growth, presenting thus many points of interest which are carefully detailed in the description.

Dr. Brinton has described and depicted the appearances of examinations of several morbid tumors of different kinds, cancerous and not cancerous. Dr. Da Costa has investigated the different forms of *epithelial* tumors. Their relative frequency in twenty-five cases he examined were—eleven of the lips, seven of the face, one of the trunk, two of the extremities, one of the penis, two of the tongue, one of the gums. Their minute structure is represented as consisting of cells, not usually larger than the ordinary pavement epithelial cells, of granules, and of small globular cells, which are either young epithelial cells, or free nuclei. The enlarged papillæ are coated with cells. The author also notes the appearance of cancer of the skin, which he believes to be distinct from epithelial formations. He gives the duration of twenty cases of epithelial growths that had come under his observation, from which it would appear that frequently the tumors are of slow growth, and the result of local irritation; and rejects the idea that they are cancers modified by the structures in which they are developed. The paper is followed by a report of several of the cases.

### PARASITES.

*Silas Durkee.*—"Sarcina Ventriculi in Connection with Disease;" Am. Journ. of Med. Science, January, 1854.

Dr. Durkee describes the presence of sarcinæ in a case of ulceration of the lining membrane of the nose. A smooth glass rod being passed up the right nostril, upon its withdrawal was found loaded with a thick white caseous substance, not unlike what is often found upon the ulcerated tonsils of a scrofulous subject. Upon subjecting this to the microscope, masses of sarcinæ ventriculi, mixed with epithelium, free epithelial nuclei, and mucus, were noted. The author then gives the history and description of sarcinæ in general, and cites the instances adduced by Jenner and Heller, which prove that they originate in other organs besides the stomach. Dr. Durkee's case is among the first reported, which establish their general diffusion. They have since been found in the brain, and by Zencker and Virchow, in the lungs.

Dr. Leidy's work on Parasites is so well known, that we need but refer to it here as one of the best monographs which have appeared anywhere on the subject of which it treats.

---

### SPECIAL PATHOLOGICAL ANATOMY.

### NERVOUS SYSTEM.

*Edward Hallowell.*—"Observations on the Prevention of Phthisis, with Cases Illustrating its Natural History;" Am. Journ. of Med. Science, January, 1850.

*W. Henry Thayer.*—"Cases of Apoplexy, and the Various Forms of Softening of the Brain;" Am. Journ. of Med. Science, July, 1851.

*Samuel Kneeland, Jr.*—"Report on Idiotic Crania, Idiocy, and Cretinism," read before the Boston Society for Medical Improvement; Am. Journ. of Med. Science, April, 1851, p. 349.

*Thomas F. Cock.*—"Cases of Cerebral Disease;" Am. Journ. of Med. Science, October, 1852, p. 371.

We have placed the paper of Dr. Hallowell under this head, because, although prefaced by some general remarks on the subject of tubercle, its main interest is derived from the narration of cases of tubercular meningitis, embracing a very complete summary of their post-mortem appearances. It is a pleasure, amid so many hurried and incomplete descriptions of autopsies, especially of the brain, as we have had to look over in preparing this record, to arrive at such accurately examined cases. In the first case, there was a considerable quantity of yellowish tuberculous matter deposited around the commissures of the optic nerves, with opacity and thickening of the arachnoid in this spot. Beneath the arachnoid, upon the upper surface of the brain, were observed a very considerable number of opake yellowish granulations; they followed the course of the vessels, and were most abundant in the fissure of Sylvius. In the second, third, and fourth cases, a yellowish deposit also existed, surrounding the commissure of the optic nerves. Upon the surface of the left ventricle, in one case, were observed a number of yellowish deposits, similar in appearance to those upon the base of the brain. Effusion into the ventricles is frequently mentioned; and, on the whole, the post-mortem examinations agree with the pathological appearances which the continental observers consider as belonging to tubercular meningitis.

Dr. Thayer illustrates the pathological difference between softening of the brain around an apoplectic clot, and that which occurs under other circumstances. The cases which Dr. Cock has detailed are worthy of a careful perusal. Especially interesting is the case of gangrene of the brain. It occurred in a person, aged twenty-two, who had had a discharge from his ear for several months. Just over the ventricles the brain was softened so much as to rupture, and permit the exudation of their contents. A dirty, greenish, sero-purulent fluid filled both cavities, and had imparted a dark-green color to the walls of the ventricles, which tint extended about a line in depth. The next incision on the right side, after opening the ventricles, revealed a dark-green mass, in size three inches by two, resembling in appearance and odor a gangrenous lung; the smell, though very fetid, was not quite as pungent as pulmonary gangrene. This dark portion had a well-defined edge, which seemed elevated, and around its border the cerebral tissue was soft.

Examinations of cases of tetanus are not so frequent as to preclude the necessity of stating the appearances in an autopsy made by Dr. E. P. Bennett* on a lad of thirteen, in whom tetanus set in after a lacerated wound of the hand. The venous circulation was deeply congested, the inner surface of the dura mater was of a deep rose-color, and in places slightly adherent to the pia mater, which was highly injected, especially the portion covering the pons varioli, medulla oblongata, and spinalis; the cord was of a rose-color. The theca vertebralis was highly injected, and adherent to the pia mater by a copious plastic effusion. The author regards this case as one of tetanus, caused by an inflammation of the medulla oblongata and spinalis.

The report of Dr. Samuel Kneeland on idiotic crania, idiocy, and cretinism, is not only interesting to the psychologist and phrenologist, but has points of interest to the pathological anatomist. It contains some extremely valuable measurements of skulls of idiots and of hydrocephalic heads, to which our limits do not permit us more than to refer.

* New York Medical Journal, vol. vi., 1851.

DIGESTIVE SYSTEM APPARATUS.

*Charles M. Allin.*—"Retro-pharyngeal Abscess, its Medical History and Treatment, with a Statistical Table of Fifty-eight Cases;" New York Journ. of Med., vol. vii., 1851, p. 307.

*John A. Lidell.*—"Upon Acute Gastritis, with Cases;" New York Journ. of Med., vol. x., 1852, p. 211.

*Jonathan Kneeland.*—"Cases of Cancer Occurring in the Abdominal Viscera, with Remarks;" New York Journ. of Med., November, 1853.

*Samuel Kneeland, Jr.*—"Cases of Fistula Communicating with the Gall-bladder;" Am. Journ. of Med. Science, April, 1855.

*F. V. L. Brokaw.*—"Dysenteric Ulceration and Sloughing of the Mucous Membrane of the Intestines as a Cause of Abscess of the Liver;" St. Louis Med. and Surg. Journ., November, 1857.

*J. Da Costa.*—"Cancer of the Pancreas;" Proceedings of the Pathological Society of Philadelphia, vol. i. p. 109; or North American Medico-Chirurgical Review, September, 1858.

*Pharynx and Œsophagus.*—In a carefully prepared paper, Dr. Charles M. Allin has discussed the formation of abscess between the posterior wall of the pharynx and the cervical vertebræ. The paper is not limited to the morbid appearances, but furnishes a complete summary of the subject in its various relations. We have introduced it here, because the table records the post-mortem phenomena, for the most part, in a very concise manner. In the chronic variety, one of the causes is scrofulous disease of the vertebræ, which, in the cervical region, is ordinarily confined to the articular surfaces. For this reason, in long-continued cases, in which the abscess has been opened and the disease has not been arrested, dislocation of the vertebræ may take place, and death be the result of laceration, or compression of the spinal cord.

*Tongue.*—An examination of cancerous disease of the œsophagus is communicated by Dr. Ware. The disease extended from the top of the arachnoid cartilage to the lower extremity of the thyroid gland.*

*Stomach.*—The occurrence of acute gastritis as an idiopathic affection is, it is hardly necessary to state, considered by all writers as exceedingly rare. Dr. Lidell has met with five instances. They all presented the post-mortem appearances which are usually admitted to belong to inflammation of the stomach, but as they were cases of sudden death, which the coroner was called upon to investigate, their histories are not so perfect as to justify us in concluding that they were all cases of acute idiopathic gastritis. They are, nevertheless, an interesting contribution to this obscure subject. A form of disease of the stomach, not common, has been noticed by Dr. J. B. S. Jackson.† The organ was contracted, thick, and fleshy to the feel externally; the disease extended to every

---

* Am. Journ. of Med. Science, April, 1854.

† Records of the Boston Society for Medical Improvement; Boston Med. and Surg. Journal, vol. lii., 1855, p. 416.

part of it, but not beyond the orifices. The muscular coat was very much thickened, but had scarcely the density of scirrhus. The submucous cellular membrane was thickened, but not uniformly so, the result of which was that the inner surface of the organ was thrown into prominences or knobs; the mucous membrane was generally dark, and at the elevated portions decidedly red. In the large curvature was an ulcer about an inch in diameter, with defined, thick, and firm edges, and penetrating through the whole thickness of the organ.

*Liver.*—The proportion of fat in a *fatty* liver* is estimated by Dr. John Bacon, Jr., as equivalent to 53·13 per cent. In the specimen examined, the whole liver contained about five pounds five ounces of fat, solid at the ordinary temperature, but melting at about 98° F., into nearly transparent oil, which became quite clear at 110° F. Stearine, margarin, and olein were present in it; probably also a little cholesterine. Dr. A. Clark† regards the character of the deposit in *waxy* liver as resulting from a new production of a peculiar nature, and is not disposed to coincide with the view of Bennett, that it is a late stage of fatty degeneration. In order to ascertain, if possible, the character of this deposit, whether it be a peculiar form of fatty degeneration, or whether the reaction of chemical agents be the same as upon animal tissue, he tested sections of the part containing the cells, with rectified sulphuric ether, in expectation of their dissolving. No change whatever was produced. He then employed boiling ether, but still no change was effected. They were next treated with strong acetic acid; this seemed to render the cells more transparent, but they remained entirely unchanged in form. Maceration with boiling alcohol produced no change, though it reduced the sections to the consistence of alcohol. Finally they were treated with nitric acid of various strength, and it was observed that when the acid was sufficiently strong to dissolve animal fibre, they did change, but when it was of less strength they did not, which proved the deposit to be of an animal nature. This opinion does not accord with one that has been since expressed by Virchow, that these waxy livers are really amyloid degenerations.

*New formations* in the liver of a fibro-plastic nature, are mentioned by Dr. Christopher Johnson.‡ *Cysts* from the liver, coughed up from the pulmonary passages, were exhibited at the Pathological Society of Philadelphia.§ Drs. Sayre and Clark have placed on record cases of melanotic *cancer* of the liver. Drs. Griscom and Levick relate‖ a very interesting case of melanotic cancer of the liver, in which also the various thoracic and abdominal organs, including the supra-renal capsules, were melanotic, without any external evidence of the disease being present.

---

* Boston Society for Medical Improvement; Am. Journ. of Med. Science, April, 1854, p. 355.

† Proceedings of the New York Pathological Society; New York Journ. of Med., September, 1854.

‡ Medical Examiner, April, 1854.

§ Proceedings, p. 35.

‖ Transactions of the College of Physicians of Philadelphia; Am. Journ. of Med. Science, April, 1858.

*Abscesses* of the liver following dysentery, are mentioned by J. T. Metcalfe.* Dr. Brokaw's paper on the subject is very conclusive. In twenty-seven fatal cases of dysentery, in which he made post-mortem examinations, there were abscesses in the liver in twenty-two. In fifteen of these cases the dysentery was of a chronic form, and the large intestines in all were more or less ulcerated and thickened. In some of the worst cases there was slight ulceration of Peyer's glands near the termination of the ilium, and the mucous membrane of the large intestines was most extensively ulcerated throughout its entire extent, while in the less aggravated cases there were only a few small ulcers in the rectum, sigmoid flexure of the colon and cæcum. In the acute form, the mucous membrane of the large intestines was in several cases almost entirely destroyed by sloughing; the abscesses were not circumscribed by lymph, were quite numerous, and varied much in size. Ten out of the twenty-two cases had suffered from intermittent fever at a time previous to their admission into the hospital with dysentery. The author adds the twenty-two cases of hepatic abscess which he has examined to the seventeen mentioned by Budd, and the twenty-nine of Annesley, and finds that in fifty-five of these sixty-eight cases there was ulceration or sloughing of the mucous membrane. The abscesses, he agrees with Budd, have their origin in contamination of the portal blood. Instances of abscesses of the liver discharging through the lungs, are mentioned by Drs. Jackson† and Morehouse.‡

*Gall-bladder.*—Dr. Morton§ has reported a case of fistula of the gall-bladder in a female aged sixty-five. A tumor formed over the liver, and opened near the twelfth rib, about four inches from the median line; the discharge continued from the fistulous orifice until her death. The gall-bladder was found firmly attached to the walls of the abdomen, and completely blocked up by gall-stones. The fistulous orifice led from the gall-bladder through the parietes of the abdomen, and opened at the point above mentioned. Dr. Samuel Kneeland, in his instructive paper on fistulæ of the gall-bladder, places on record several cases of fistulæ of the gall-bladder, and then examines into those which have been reported in the different periodicals. The opening in a case he had under observation, had irregular edges, and was situated about two and a half inches above, and four inches to the right of the umbilicus. Seven calculi were passed through it, composed principally of cholesterine. A rupture of the gall-bladder occurred in the practice of Dr. S. H. Freeman.||

*Intestines.*—Attention is directed by Dr. Jackson¶ to an affection of the internal follicles in young children who have died of acute diseases, such as convulsions, the exanthemata, croup, and pneumonia. The swelling and redness are especially marked in the glands of Peyer.

---

* Proceedings of Path. Society of New York; New York Journ. of Med., vol. iv., 1850; Proceedings of Path. Society of New York. 1857.

† Boston Medical and Surgical Journal, January, 29, 1857.

‡ Proceedings of Pathological Society of Philadelphia, vol. i. p. 56.

§ Proceedings of Pathological Society of Philadelphia, p. 5; North American Medico-Chirurgical Review, January, 1858.

|| Boston Medical and Surgical Journal, November 26, 1857.

¶ Boston Society for Medical Improvement; Am. Journ, of Med. Science, April, 1852, p. 352.

In a patient who died after one week's illnes, Dr. Storer* discovered gangrene of the appendix cæci, which contained within its cavity an oblong oval mass of hardened fæces, with several short pieces of hair running through it ; no perforation had taken place. Another instance of gangrene of the vermiform appendix is related by Dr. J. M. Warren; the appendix vermiformis was found perforated at both ends, and contained in its central portion, a mass of indurated fæces. The appearances of the gangrenous part are unfortunately not particularly described, and room exists for doubt as to the destruction of tissue really having been gangrenous, or as to the cases being different from the ordinary ulceration of the process met with, and such as we find noticed by Dr. Sands† and other practitioners.

Dr. F. F. McElrath‡ describes a cancerous tumor of the cæcum, embracing portions of the ascending colon and of the ileum.

*Pancreas.*—In Dr. Jonathan Kneeland's remarks on cancer of the abdominal viscera will be found the description of a case of cancer of the pancreas. Further cases have been reported by Drs. Da Costa,§ Agnew,‖ and Demme.¶ The former has also, in a paper on cancer of the pancreas, given its morbid anatomy and clinical history, based on the recorded cases of this affection.

Concretions have been found in the pancreatic duct by Dr. McCready,** consisting of mixed phosphate and carbonate of lime. The mass was over one-third of an inch in diameter, and above an inch long. The patient died of albuminuria. Simple induration of the organ, resembling malignant disease, is described by Dr. Clark ;†† an interesting lesion of the pancreas produced by a gall-stone obstructing the pancreatic duct, by Dr. Pepper.‡‡

Among the affections of the *peritoneum* we shall merely cite here an encephaloid tumor attached to the peritoneum and to the vertebræ, weighing thirty pounds, recorded by Dr. Watkins.§§

### CIRCULATORY APPARATUS.

*Bennett Dowler.*—" Fragmentary Notes on Pathological Anatomy ;" New Orleans Med. and Surg. Journ., September, 1856, continued to several numbers of 1857.

---

* Boston Society for Medical Improvement; Am. Journ. of Med. Science, January, 1581; Ibid., October, 1851, p. 368.

† American Medical Monthly, October, 1857, p. 238.

‡ New Orleans Medical Journal, May, 1858.

§ Proceedings of Pathological Society of Philadelphia, p. 8.

‖ Ibid., p. 84.

¶ Medical and Surgical Reporter, November, 1858,

** Proceedings of Pathological Society of New York; N. Y. Journ. of Medicine, January, 1856.

†† Ibid., September, 1854.

‡‡ Am. Journ. of Med. Science, January, 1857.

§§ New York Journal of Medicine, vol. v. p. 337; Proceedings of New York Pathological Society.

*Heart.*—Dr. Bowditch* has met with a rare form of cardiac disease—concentric hypertrophy. An unusual affection of the valves consisting in a slit in the pulmonary valves, is described by Dr. A. Clark.† One of the folds of the valves was torn in two places; the lesion did not seem to be recent. A firm clot had insinuated itself into these openings, and completely filled them. A fatty condition of the heart is described by Dr. Macgibbon,‡ in which free oil is stated to have been found in the blood. The blood corpuscles under the microscope, were much broken down. Air also was present in the blood. Unfortunately it is not definitely stated that the blood, besides that in the heart, yielded oil, and it is by no means certain, from the report, that the oil was not derived from the heart itself during the examination, as might so easily have happened. An instance of cancer of the pericardium and heart is recorded by Dr. Ellis.§

A specimen of dilatation of the left ventricle at one point, is described by Dr. Jackson|| as a true aneurism of the heart. A slight enlargement only could be perceived externally. Internally the sac was of a circular form, measured about three inches in diameter, and contained coagula that had evidently formed before death. The muscular substance was yellowish-white, and was found to be fatty. The same author¶ describes two specimens of malformations of the heart, in one of which an opening existed between the two auricles, more than half an inch in diameter.

Dr. Carter P. Johnston** has figured a heart in which the aorta took its origin from the right ventricle, and the pulmonary artery from the upper and posterior portion of the cavity of the left ventricle by a free and patulous orifice. With the exception of its orifice, and the consequent changes produced in the first portion of its course, the distribution of the aorta was normal. The child had been cyanosed to a greater or less extent from its birth. A heart with an interventricular opening is described by Dr. Cabot.†† The organ was obtained from an adult twenty years of age. The opening between the two ventricles was sufficiently large to allow the tips of the two fingers to pass through. The pulmonary artery had two well-developed valves. The passage to it, from the right ventricle, to the extent of half an inch or more from the free edge of the valves, was very much contracted. Dr. Darrach also describes a heart in which the two ventricles communicated.‡‡ Dr. Leigh§§ speaks of a specimen in which but one auricle and one ventricle existed, with a well-developed valve between the

* Boston Society for Medical Improvement; Am. Journ. of Med. Science, July, 1853.

† Proceedings of the New York Pathological Society; New York Journ. of Med., September, 1854.

‡ Am. Journ. of Med. Science, January, 1856.

§ Boston Society for Medical Improvement; Boston Med. and Surg. Journal, October 2, 1856.

|| Am. Journ. of Med. Science, April, 1850, p. 369.

¶ Ibid., p. 370.

** Ibid., October, 1850, p. 370.

†† Ibid., April, 1854, p. 352.

‡‡ Proceedings of Pathological Society of Philadelphia, p. 53.

§§ Am. Journ. of Med. Science, p. 355.

two cavities. Dr. White* depicts a heart taken from a child thirteen months of age, in which, accompanying an almost fœtal condition of the organ, the interventricular septum was wanting at its upper portion, and the origin of the aorta was situated immediately over it, so as to receive the blood from both ventricles.

*Aneurism.*—Two cases of aneurism of the aorta, terminating by rupture into the trachea, are reported by W. H. Van Buren.† Intercurrent bronchitis existed in both; in one, nothing but the signs of this were, during life, elicited. The arch of the aorta was, in this case, firmly attached to the trachea over a space nearly an inch in diameter. Two ulcerated openings communicated directly with the cavity of the aneurism. They were partly blocked up by coagula, evidently of long standing. We may mention among the more interesting of the recorded cases of aneurism, one bursting into the trachea, reported by Dr. Minot;‡ into the bronchial tubes, by Dr. Forbes;§ cases rupturing externally, recorded by Drs. Coale‖ and Packard;¶ bursting into the œsophagus, by Dr. McCready.** In Dr. Packard's case, pieces of roughened bone were found within the cavity of the aneurism, evidently remains of the sternum. They were irregular in shape, and eroded. The end of the right clavicle, and the upper two ribs on the same side, were in a similar condition, and projected through the wall of the sac; both sterno-clavicular articulations were absorbed. The sternum above the second rib was entirely gone, except a slender strip which had formed the border on the left side.

*Veins.*—A very interesting case of ossification and obliteration of the ascending vena cava is detailed by Dr. G. L. Collins.†† The descending vena cava was about the usual size, and distended with blood. The ascending vena cava was of the usual size, and healthy to a point near the origin of the right renal vein, when it became at once impervious, terminating abruptly in a sort of cul-de-sac. Below this point, the vein, for two and a half inches, was converted into a firm cord about two lines in diameter, in the substance of which was a deposit of compact bone, sufficiently firm to arrest the passage of the knife. The vena cava from this ossific deposit to its bifurcation, was converted into a dense impervious cord, as were the common iliac veins, though the latter retained a trace of the tubular form, as a small probe could be passed through them. The patient, a man aged thirty-four, had in addition to this disease of the veins, a large vascular tumor, which occupid a position behind, and a little to the left of the rectum. Dr. Bennett Dowler, in his notes on Pathological Anatomy, and fragmentary notices of the most various lesions and condition, commented upon in the author's peculiar style, forcibly draws attention to the occurrence of air in

* Buffalo Medical Journal, July, 1855.

† Proceedings of New York Pathological Society; New York Journal of Medicine, vol. v., 1850.

‡ Am. Journ. of Med. Science, July, 1850, p. 29.

§ Proceedings of Pathological Society of Philadelphia, vol. i. p. 64.

‖ Am. Journ. of Med. Science, July, 1851, p. 75.

¶ Ibid., July, 1858.

** New York Journal of Medicine, March, 1856, p. 267.

†† Am. Journ. of Med. Science, October, 1850, p. 391.

the veins and in different organs, wholly independent of post-mortem putrefaction and traumatic lesions.

*Obstruction of the Deep Jugular Veins.*—Dr. Metcalfe* relates an instructive case caused by pressure from enlargement of the lymphatic glands. The glands commenced to enlarge after measles, until a swelling, which occasioned intense dyspnœa, extended from the clavicle, on the right side, to the vertex; this steadily increased until the child died. On raising, post-mortem, the skin of the right cervical region, the whole of the subjacent parts were seen to be firmly matted together by fibrous effusion. Where the deep jugular vein was pressed upon, a clot of fibrin was found obstructing the flow of blood in this vessel.

*Lymphatics.*—Dr. Thomas Gage narrates† the history of a patient in whom the sole lesion, after death, was found to consist in primary encephaloid disease of the lymphatic glands of the abdomen. All the organs of the thorax and abdomen were perfectly healthy.

DUCTLESS GLANDS.

*Jas. J. Waring.*—"Report of Three Cases of Ruptured Spleen, with Remarks on the Different Organic Changes which give rise to this Lesion, and the Different Modes in which it may occur;" Am. Journ. of Med. Science, October, 1856.

*Isaac E. Taylor.*—"On Supra-renal Capsular Disease;" New York Journ. of Medicine, September, 1856.

*Spleen.*—Dr. Theophilus Mack‡ describes a rupture of the spleen from what is supposed to have been sudden hyperæmia; the appearances of apoplexy were found in the organ. The whole subject of rupture of the spleen is treated in its various relations by Dr. Jas. J. Waring. In one of the cases he reports, the spleen was healthy, and simply engorged; while in two others the organ was of unusual size and thickness, extremely firm in structure, and remarkably brittle. An instance of enlargement of the liver and spleen, with concrete pus in the heart, (leucocythæmia?) is mentioned by Dr. Bowditch.§ The blood in a case of enlargement of the spleen was examined by Dr. Addinell Hewson.‖

*Supra-renal Capsules.*—The post-mortem appearances in several instances of disease of the supra-renal capsules, have been described by American practitioners; among them we may mention a case by Dr. Gould,¶ and an interesting paper, in which several cases are recorded by Isaac E. Taylor.

---

* Proceedings of New York Pathological Society, in New York Journal of Medicine, 1850, vol. i. p. 37.

† Boston Society for Medical Improvement; Am. Journ. of Med. Science, April, 1854.

‡ Am. Journ. of Med. Science, January, 1857, p. 27.

§ Ibid., July, 1857.

‖ Ibid., October, 1857.

¶ Boston Med. and Surg. Journal, July 16, 1857.

### DISEASES OF THE RESPIRATORY ORGANS.

*W. Parker.*—"Polypus Laryngis;" New York Journ. of Med., vol. vii. 1851.

*J. Forsyth Meigs.*—"Remarks on Atelectasis Pulmonum, or imperfect Expansion of the Lungs, and Collapse of the Lungs in Children;" Am. Journ. of Med. Science, Jan. 1852.

*J. Da Costa.*—"An Inquiry into the Pathological Anatomy of Acute Pneumonia;" Am. Journ. of Med. Science, Oct. 1855.

*Henry J. Bowditch.*—"Peculiar Case of Diaphragmatic Hernia, to which is added an Analysis of most, if not all, of the Cases of Diaphragmatic Hernia found recorded in the Annals of Medical Science;" BuffaloM ed. Journ., June, 1853.

*Larynx.*—Dr. Parker, in giving the history of two cases of polypus of the larynx, describes their structure, and alludes to similar cases noticed by authors. One attached to the epiglottis is reported by Dr. H. Green;* in another case the microscopical appearances are stated, by Dr. W. J. Burnett,† to have consisted of fibrous tissue, and large numbers of pavement epithelial cells folded in it. In both of Dr. Parker's cases the polypi caused death by suffocation. One was of the kind designated as mucous polypus; the other consisted in a cluster of warty excrescences, which, doubtlessly, if examined with the microscope, would have shown epithelial formations of the same structure as in the specimen investigated by Dr. Burnett.

An unusual form of laryngitis is mentioned by Dr. Jackson.‡ The entire lip of the glottis, upon the left side, was very much swollen, fleshy to the feel, and of a bright-red color, except upon the summit, where there was a defined, brownish, soft slough, three or four lines in diameter. The subjacent tissues were either of a deep-red color or yellow from the infiltration of something resembling very thick pus. The lip of the glottis upon the other side was perfectly healthy. A cancerous tumor of the larynx is reported by Dr. Bowditch.§ It was the cause of death, and was found to occupy the right ventricle of the larynx, and to project downward into the trachea about half an inch. It had arisen in the cellular structure between the cartilages, but had not involved them. Further instances of laryngeal diseases will be found mentioned in the various contributions to the subject which Dr. Horace Green, of New York, has, of late years, published.‖

*Lungs.*—Dr. Meigs's Essay on Atelectasis and Collapse of the Lungs, although more a clinical than a pathological paper, embraces the description of several post-mortem investigations of this affection, and may be regarded as an interesting addition to this as yet unsettled subject.

---

* New York Journ. of Med., vol. vi. p. 22.

† Am. Journ. of Med. Science, July, 1855, p. 37.

‡ Am. Journ. of Med. Science, p. 367, April, 1850.

§ Boston Med. and Surg. Journ., May, 1858.

‖ Among them "Treatment of Polypi of the Larynx;" New York, 1852.

Dr. Da Costa has investigated the morbid anatomy of acute pneumonia. He describes the lung as seen in the first stage, and depicts its appearance under the microscope. It does not differ much from congested lungs, excepting in containing numbers of exudation corpuscles. He concludes, from chemical analyses of lungs which were congested, that, in the stage preceding red hepatization, the amount of fat is increased. As the result of minute examinations, he states that the exudation is almost entirely confined to the air-cells, and regards the third stage, not as it is commonly supposed, as a suppuration, but believes that, in many cases, it is a gradual breaking up of the exudation, and secondarily of the lung tissue, allied perhaps to fatty degeneration, but not very frequently of a suppurative nature. A question requiring to be solved, and which he answers in the affirmative, is, "May a variety of exuded lymph give to the eye the peculiar yellowish appearance of the third stage, and yet not have been preceded by red hepatization?" He believes that cases of this yellow condensation occur, which form a distinct pathological species of consolidation, and which have been incorrectly classed as the third stage of pneumonia. Under the microscope they exhibit no elements indicative of suppuration, indeed slight traces of any degeneration, but distinct nucleated cells of varying shapes, and a homogeneous basis substance in which the cells are imbedded.

An instance of very extensive abscess of the lung is recorded by Dr. W. Spencer.*

Morbid growths in the *pleura* are mentioned by Dr. Woodward in the paper above alluded to, while a large fibro-nucleated tumor filling the left pleural cavity is well described by Dr. Storer.†

Of the more uncommon diseases of the thorax, we may refer to Dr. Finnell's specimen of *diaphragmatic hernia.*‡ The stomach was found, in the left pleural cavity, pushing up the lung as high as the third rib, and crowding the heart to the right side. In Dr. Bowditch's complete and elaborate paper will be found a most interesting instance of a diaphragmatic hernia which had existed in a man who died at the Massachusetts General Hospital, with fracture of the spine, caused by a heavy blow upon it. The whole left side of the diaphragm was found wanting. The stomach and a great part of the intestines lay in the left pleural cavity, compressing the left lung, and forcing the heart to the right side of the sternum. The analyses of cases recorded by authors, which is added to the description, makes this contribution extremely valuable.

---

### URINARY ORGANS.

*Stephen Smith.*—"Contribution to the Statistics of Rupture of the Urinary Bladder, with a Table of Seventy-eight Cases;" New York Journ. of Med., vol. vi., 1857.

---

* Cincinnati Medical Observer, August, 1857.

† Boston Med. and Surg. Journ., May, 1858.

‡ Proceedings of Path. Society of New York; New York Journ. of Med., Jan. 1856, p. 81.

*William J. Burnett.*—"The Microscope and Renal Affections, and particularly that Condition of the Kidney known as Bright's Disease;" Am. Journ. of Med. Science, October, 1851, p. 373.

*Charles Fricke.*—"Remarks on the Relation of the Dumb-bell Crystals to Uric Acid;" Am. Journ. of Med. Science, July, 1850.

*John Bacon, Jr.*—"Observations on the Dumb-bell Urinary Deposit;" Am. Journ. of Med. Science, April, 1851.

*John C. Dalton, Jr.*—"On a New Form of Phosphate of Lime in Crystals, as it occurs in Putrefying Urine;" Am. Journ. of Med. Science, April, 1851, p. 368.

*John T. Plummer.*—"On Dumb-bell Forms in Urine;" Am. Journ. of Med. Science, Oct. 1853.

*George W. Miltenberger.*—"Creatine Spontaneously Deposited in Oxaluria;" Med. Ex., Jan. 1855.

*J. Cheston Morris.*—"Creatine in the Urine;" Med. Ex., Sept. 1855.

*S. Weir Mitchell.*—"Observations on the Generation of Uric Acid, and its Crystalline Forms;" Am. Journ. of Med. Science, p. 121, July, 1852.

*John Bacon, Jr.*—"Urinary Deposit of Epithelial Nuclei;" Am. Journ. of Med. Science, Oct. 1852.

*Benjamin S. Shaw.*—"Crystals of Urate of Potassa in Urine;" Am. Journ. of Med. Science, Oct. 1853, p. 381; and "A Peculiar Form of Uric Acid in Urine;" Ibid., p. 382.

The affections of the urinary organs and the urine have received considerable attention from American writers. In addition to the many papers, some of them very valuable, which we have just cited, much interesting information may be obtained on the subject of morbid alterations of the urinary organs from several chapters in the work of Dr. Fricke, and from the Treatise on the Diseases of the Urinary Organs, by Dr. Gross. A volume on Urinary Affections has also recently been published, by Dr. Morland, of Boston. We may further refer with some pride, to the many excellent communications on the subject of calculi, which the surgeons of this country have made to the profession.

*Kidney.*—On the field of urinary pathology we meet again with Dr. W. J. Burnett, whose name we have had occasion to introduce more than once, and whose early death has occasioned that profound regret always experienced when one arduously devoted to science is cut short in a career which promised to be so brilliant. Dr. Burnett describes *Bright's disease* as decidedly inflammatory; it is primitively, he thinks, an acute or subacute nephritis, and of a uniform character. His remarks are on the side of those who believe in the unity of Bright's disease, and throughout his essay he takes an opportunity of inculcating what every practical pathologist must admit, how unreliable is the examination of the kidney with the naked eye. Some of the cases illustrate the occurrence of fatty liver with Bright's disease, although the kidneys were almost free from fat; and also of fatty kidney without Bright's disease; and the author takes, therefore, decided ground against those who think that the different appearances witnessed in Bright's disease are indicative of separate and distinct affections.

Of the rarer forms of disease of the kidney, we may cite a well-marked instance of cancer of the kidney, reported by Dr. J. C. Foster.* The kidney, in a case reported by Dr. Busch,† communicated with the external parts, owing to the presence of renal calculi, which were discharged through the lumbar region.

A rare malformation of the kidney, single kidney, is described by Dr. Morton, and interestingly commented upon by Dr. Harris.‡

*Bladder.*—A specimen of gangrene of the bladder was exhibited by Dr. Hall, at a meeting of the Pathological Society of Philadelphia, October 20th, 1857.§ The gangrene followed over-distension of the organ, which was found very much dilated, and filled with decomposed intensely alkaline urine, mixed with blood. The mucous membrane was soft and of a dark-red color; at one portion were several striated eschars, and around these small depressions; the membrane was of a greenish-gray or bronzed color; here and there were spots of ecchymosis.

Dr. Smith has collected together, in an elaborate paper, seventy-eight cases of rupture of the bladder, including the one published by Dr. Kneeland.|| We must refer to the paper itself for the morbid appearances. They are tabulated, and thus easy of reference. By far the most frequent seat of rupture is in the posterior walls. Cancer of the bladder is described by Dr. Jackson¶ and by Dr. Hodges.**

*Urine.*—Dr. Fricke, in the paper quoted, differs from Golding Bird, as to the composition of dumb-bell crystals, and comes to the conclusion that they are not, as has been stated, oxalate of lime, but really transformed crystals of uric acid. "If," says our author, "oxalic acid and lime be added together, the crystals formed under these circumstances are always double pyramids, although at times so small as to be scarcely perceptible; and however long the deposit be allowed to remain, neither dumb-bells nor ovals are ever found to make their appearance. If a deposit, consisting entirely of uric acid, be carefully washed, and clear water added, we may discover, in a certain number of cases, after the lapse of a few days, that dumb-bells are present, indicating, beyond a doubt, their formation from uric acid without the addition of lime."

Dr. John Bacon, on the other hand, in an elaborate and exceedingly valuable paper, has proved, by chemical experiments, that the dumb-bells consist of a salt of lime, convertible by a moderate heat into the carbonate, and that the lime is combined either with oxalic acid, or an organic acid nearly related to it.

---

* Proceedings of Path. Society of New York; New York Journ. of Medicine, Jan. 1853, p. 59.

† Proceedings of Path. Society of New York; Am. Med. Monthly, Sept. 1857, p. 184.

‡ Proceedings of Path. Society of Philadelphia, vol. i. p. 79.

§ Proceedings, p. 6, vol. i., or North American Medico-Chir. Review, January, 1858.

|| New York Journ. of Med., March, 1851.

¶ Boston Med. and Surg. Journ., Jan. 1857.

** Ibid., May, 1858.

He also proves that properly prepared oxalate of lime is soluble in strong acetic acid, and that it, when rapidly deposited from solution, affords zeolitic forms similar to those obtained from the dumb-bells. By the action of polarized light these latter exhibit concentric-colored bands, traversed by a dark cross. Although the octohedra of oxalate of lime do not act on polarized light, the prismatic crystals of this salt, which abound in various plants, possess strong polarizing power. He regards the dumb-bells as made up of very slender prisms, and not of lengthened octohedra, as Dr. Bird supposes, and is inclined to the belief that the oval bodies which this observer has described, are dumb-bells seen endwise; they show with polarized light one or two almost circular rings near the centre, and an oval band near the outside. The conclusion of this valuable paper, while it refutes the opinion of Dr. Fricke, strengthens the views of Golding Bird, as the dumb-bell crystals are stated to consist "of a salt of lime, containing either oxalic, oxaluric, or perhaps some other organic salt easily converted into the oxalic; but the exact nature of the acid remains to be determined by future investigation."

Dumb-bell forms, hovever, have been found in the urine, which were, as far as the chemical tests indicated, not oxalates. In the urine of a man crippled with chronic rheumatism, Dr. Plummer saw dumb-bell crystals, which, on chemical analyses, he believed to have been, unquestionably, sulphates. In the course of his experiments oxalate of soda dumb-bell crystals were artificially produced.

Dr. Miltenberger's observations have demonstrated the occasional presence of creatine in the urine of persons suffering from oxaluria; and Dr. Cheston Morris testifies to its occurrence in a man who had suffered under the phosphatic diathesis for nearly three years.

Dr. Fricke* speaks of a specimen of black urine.

Dr. John C. Dalton has described radiated crystalline bundles of phosphate of lime in urine that has been standing for some days.

Dr. Mitchell, in his paper on uric acid, notices the various forms of this substance, especially the larger-shaped crystals. He speaks especially of the curves on the lozenges, which he regards as furnishing the key to those upon the tabular variety, and comes to the conclusion "that these are in fact large lozenges, of no great thickness, but having an oblong or square surface, on which are seen the marks of their primitive form, and around which the circular crystals have collected, clothing it completely." A peculiar form of uric acid crystals, and also of crystals of urate of potassa is depicted by Dr. Shaw.

Dr. John Bacon describes a urinary deposit consisting of epithelial nuclei. The deposit formed in the urine has some resemblance to a purulent sediment; but is darker and more readily diffused. The urine was voided by a patient in the early stage of albuminuria. The bodies are nearly, if not quite, spherical; their surface is generally smooth, and they contain several dark granules with bright centres. They are less transparent than pus globules, and not affected by acetic and nitric acid.

*Urinary Calculi.*—Among the rarer forms of urinary calculi we have met with in looking over the different medical periodicals, we may cite the stel-

* Proceedings of the Baltimore Path. Society; Virginia Med. Journ., Feb. 1857.

late calculi depicted by Drs. Mitchell* and Darrach;† a large calculus, with a head of wheaten-straw for a necleus, recorded by Dr. W. H. Van Buren;‡ and one with a hair-pin for a nucleus, by Dr. Wheeler.§

### GENITAL ORGANS.

*Scrotum.*—Dr. J. M. Warren|| exhibited, at the Boston Society for Medical Improvement, four tumors removed from the scrotum of a man fifty-six years of age. They had been growing for twelve years, and on a careful examination were found to have a fatty structure, and to embrace the spermatic cord; the vas deferens was traced in its whole length passing through the centre of the mass.

A bony tumor of the scrotum taken from a Chinese, has been described by Dr. Kerr.¶ The mass closely resembled the upper maxillary bones placed in apposition, and on minute examination was found to contain bone corpuscles.

An ossified hydrocele sac was presented by Dr. Finnell to the Pathological Society of New York.** Dr. Ellis†† has met with calcareous concretions in the scrotum, which, on analysis, were found to consist of phosphate and carbonate of lime, with considerable organic matter. An enormous cancerous *testicle*, weighing six pounds six ounces, is mentioned by Dr. J. A. Swett.‡‡

*Ovaries.*—Professor Gilman§§ describes a specimen of ovaritis removed from the body of a patient fourteen days after the occurrence of puerperal mania. The symptoms of the mental affection commenced with indications of derangement of the genital functions. No disease was discovered excepting enlargement and intense congestion of the right ovary. A similar case was communicated to Prof. Gilman by Dr. Barker of Connecticut.

In an ovarian cyst Dr. Hooker|||| has met with teeth, six in number, and set in a piece of bone about three-fourths of an inch in length. The soft parts immediately about the bone closely resembled the gum; the surface was covered with epithelium. Dr. J. L. Jones¶¶ describes hair in an ovary which had been transformed into a calcareous mass. A colloid disease of the right ovary is reported by Dr. Perry,*** and was microscopically examined by Dr. H. J. Bigelow.

---

* Proceedings of Path. Society of Philadelphia, p. 27, or North American Medico-Chir. Review, March, 1858.

† Ibid., p. 31.

‡ Proceedings of Path. Society of New York; New York Journ. of Med., 1850, vol. iv. p. 323.

§ Am. Journ. of Med. Science, April, 1853. || Ibid., April, 1850.

¶ North American Medico-Chirurgical Review, January, 1858.

** American Medical Monthly, February, 1857, p. 113.

†† Boston Med. and Surg. Journal, June 18, 1857.

‡‡ New York Journal of Medicine, 1850, p. 35.

§§ Proceedings of the New York Pathological Society; New York Journal of Medicine, 1851, vol. vi. p. 41.

|||| Am. Journ. of Med. Science, April, 1854, p. 361.

¶¶ New Orleans Med. News, February, 1858.

*** Am. Journ. of Med. Science, January, 1855, p. 50.

It is not necessary to notice further the affections of the female organs of generation, as they will be fully reported upon in the report on obstetrics and diseases of women: we would merely indicate in passing, a specimen of *imperforate vagina* occurring in an unmarried female, detailed by Dr. J. B. S. Jackson.* The uterus and vagina were immensely distended. A fleshy mass of about half an inch in thickness separated the vagina from the vulva, and was the cause of the occlusion. The vagina was found cancerous by Dr. Minot.† Dr. Markoe‡ has described a fibrous cellular tumor of the anterior wall of the vagina.

---

### BONES AND JOINTS.

*Wm. S. Bowen.*—"Cases of Softening of Osseous Tissue, with Remarks;" New York Journal of Medicine, vol. v., 1850.

*Buckminster Brown.*—"Report of a Case of Extensive Disease of the Cervical Vertebræ, with Remarks on this and some other Forms of Carious Disease of the Spine;" Am. Journ. of Med. Science, January, 1853.

*Louis Bauer.*—"A Critical Examination of a Pathological Specimen of Softening of the Inter-vertebral Cartilages;" New York Journal of Medicine, May, 1854.

*Thomas M. Markoe.*—"Chronic Sinuous Abscess of Bone;" New York Journal of Medicine, May, 1858.

Dr. Bowen has described several specimens of softening of bones of the leg and of the foot. The bones were so much softened and altered in structure, as to admit of easy division with the scalpel. A section liberated a large quantity of oil. In one of the tarsal bones this was very prominent; there was rarefaction of the cellular structure of the bone, and the enlarged cells were filled by a deposition of fatty matter, leaving thus but a very thin crust of sound bone. The cartilages were not in any of the cases materially affected. Dr. Bowen believed that the bones were chemically unaltered, and that the softening was due to mere attenuation, and perhaps to oily maceration. But as the bones were not minutely examined, this point cannot be regarded as certain. It is very probable that a microscopical examination would have shown a degeneration of the bone structure.

An instance of necrosis of the tibia, followed by spontaneous fracture, is given by Dr. Charles D. Smith.§ The disease had been partly removed, but recurred after syphilis and mercurialization. Specimens of osteoid cancer are described

---

* Am. Journ. of Med. Science, July, 1850, p. 27.

† Ibid., July, 1854, p. 88.

‡ Proceedings of Pathological Society of New York; American Medical Monthly, July, 1858.

§ Proceedings of Pathological Society of New York, in New York Medical Journal, vol. v. p. 337.

by Dr. H. J. Bigelow.* Dr. A. Clark† presented to the Pathological Society of New York several cancerous bones removed from the same patient. The vertebral column was softened from cancerous disease, from the first dorsal vertebra to the promontory of the sacrum; so were the head and upper part of both femurs.

Dr. Buckminster Brown's case of disease of the cervical vertebræ is as interesting to the physiologist as it is to the pathologist. In a young mulatto, in whom there was total inability to move the head independently of the body; in whom paralysis came on gradually and late in the disease, with extreme emaciation and rigid contraction of the muscles of the extremities; and in whose neck one of the spinous processes had disappeared from its normal position, was found, after death, on the anterior face of the bodies of the cervical vertebræ, the cyst of an old abscess, containing no fluid, but communicating with the rachidian canal between the dura mater and the arachnoid, through an opening formed by the removal of the body of the second vertebra, and which extended from the first to the third. On cutting into the upper part of the cyst, toward the medulla oblongata, a loose piece of carious bone, the size of half a filbert, rolled out of the medullary cavity; another, and larger piece was subsequently found. They were the remnants of the odontoid process, and of the body of the axis, which was almost entirely destroyed. Ascending to the atlas, the disease had destroyed both inferior articulating cartilages, and partially the processes; it had extended anteriorly round the condyles, upward toward the superior condyles, and posteriorly through the left lamina of the posterior arch, breaking entirely through it at one point, and continuing on until it involved the posterior tubercle. In the occipital bone, the right articulating condyle and the basilar process were roughened, and thinned. The occipito-axoid ligament which incloses the odontoid process, was ulcerated through, thus permitting the fragments of this process to find their way into the vertebral column. Softening was further found in most of the cervical vertebræ, and in the medullary substance, from the foramen magnum to the first dorsal. The upper part of the marrow was reduced to a pultaceous, semifluid mass.

A specimen of very extensive disease of the vertebræ is described by Drs. Louis Bauer and Barthelmess.‡ It showed tubercular deposits in the bodies of the thoracic vertebræ, from the fourth to the eleventh. The greatest part of the eleventh dorsal and second lumbar vertebræ, as well as the first lumbar vertebra, had entirely disappeared. The first-mentioned of these authors also reports a very interesting case, tending to confirm the occurrence of idiopathic disease of the inter-vertebral cartilages. These presented, under the microscope, unmistakable marks of textural disintegration and fatty degeneration, but there was no evidence of tubercular matter.

One of the most singular instances of physiological pathology is furnished by Dr. Toland's§ case of whitlow, in the reproduction of bones and joints after

---

* Am. Journ. of Med. Science, July, 1852, p. 80.

† New York Journ. of Med., March, 1853.

‡ New York Journ. of Med., January, 1854.

§ Charleston Med. Journ. and Review, November, 1857, and July, 1858.

their removal. The whole subject of abscess of bone is well handled by Dr. Markoe, both in its surgical and pathological bearings.

*Affections of the Joints.*—Dr. W. H. Van Buren* has described a deformity resulting from an absorption of the head of the femur. The remains of the cervix were worn smooth on its surface, from contact with a newly-formed substitute for the acetabulum, situated on the dorsum of the ileum, beneath the glutæus minimus muscle which, with the cellular tissue, and the other muscles in its vicinity, had formed a perfect capsule for the new joint. The new acetabulum was not furnished with any coating of articular cartilage, but the portion of the ilium composing it was exceedingly smooth, and was rendered deeper by the deposit of bone around its circumference. The original acetabulum, although diminished in size and altered in shape, still retained its incrusting cartilage and its capsular ligaments.

Descriptions of eburnation of the cartilaginous surfaces of the knee-joint are given by Drs. H. J. Bigelow,† Packard,‡ and Brinton.§ Dr. A. S. Sawyer|| describes an osteoid growth connected with the capsular ligament of the right hip-joint.

---

### SKIN, AND ORGANS OF SENSE.

*Henry H. Smith.*—"Case of Molluscum developed by an Injury, and presenting, under the Microscope, the Characters of Medullary Cancer;" Am. Journ. of Med. Science, Oct. 1851.

*Waldo J. Burnett.*—"Case of Cheloidea, with some Remarks on Cheloid Growths;" Am. Journ. of Med. Science, Oct. 1853.

Neither in the anatomy of the affections of the skin, nor in the examinations of the organs of special sense, have we many observations to record. The one presents a wide, and, with the exceptions of the labors of Wilson, Simon, and Hebra, as yet an uncultivated field, in its minute details. The researches in this country on the organs of special sense have been chiefly confined to the eye, and will be found in the various American editions of the standard text-books, such as of Lawrence and Mackenzie. Of the rarer instances of affections of the *eye* which the journals record, we may mention a specimen of ossification of the retina described by Dr. Isaacs;¶ also, instances recorded by Drs.

---

* Proceedings of the New York Pathological Society; New York Journal of Medicine, 1850, vol. iv. p. 337.

† Am. Journ. of Med. Science, April, 1852, p. 353.

‡ Proceedings of the Pathological Society of Philadelphia, vol. i. p. 15.

§ Ibid., p. 75.

|| Am. Journ. of Med. Science, January, 1858.

¶ Proceedings of Path. Society of New York; New York Journ. of Med., 1851, vol. vii.

Bethune* and Gay, of melanosis of the eyeball; and cases of malignant disease of the eyeball, by Dr. Williams† and James Bolton.‡

An epithelial tumor of the cornea is reported by Dr. Bethune,§ while Dr. John H. Dix|| depicts a fibro-plastic polypus of the iris, composed almost entirely of elongated fibro-plastic cells and nuclei.

*Skin.*—The interesting case of *molluscum*, which Dr. Henry H. Smith details, came on after an injury to the skin of the forearm. At first the tumors were confined to the arm, which was amputated, but subsequently they were observed scattered over the body. A microscopical examination made on one of the tumors of the head, removed post-mortem, proved them to be medullary cancer. The disease, in this instance at least, may be viewed as an indication of a marked cachexia, giving rise to the extensive formations of cancerous tumors in the skin.

Dr. Burnett speaks of the microscopical appearances, and of the occurrence of cheloid growths. He found a specimen, which he carefully examined, to consist exclusively of fibro-cellular tissue, which had involved, during the growth and expansion of the disease, a greater or less amount of epithelium—this last appearing in the form of broken-down cells and scales. "In an anatomical point of view, then, this disease is only a fibrous epigenesis of the skin."

---

### AFFECTIONS OF VARIOUS AND UNCERTAIN SEATS.

*Lewis Smith.*—"Analyses of One Hundred and Thirty-one Cases of Hydrophobia;" New York Journ. of Med., Jan. 1856.

Dr. Smith, in his careful essay on *hydrophobia*, records the post-mortem appearances that have been noticed by different pathologists, but unfortunately they do not throw much light on this strange disease. In a case examined by Dr. Turner,¶ the cerebro-spinal fluid that escaped freely from the foramen magnum was thick and opake like pus; and the investing membranes of the medulla spinalis were seen coated with lymph throughout nearly their whole extent. The lymph was deposited more abundantly on the posterior than on the anterior surface of the cord.

---

### CHOLERA.

"Report of the Committee of the College of Physicians of Philadelphia, appointed to Examine into the Condition of Mucous Membrane of the Intestinal Canal in Persons Dying of Cholera;" Transactions of the College of Physicians of Philadelphia, No. i. vol. iii.

---

* Boston Society for Medical Improvement; Am. Journ. of Med. Science, Jan. 1853.

† Ibid., July, 1853, p. 73.

‡ Boston Med. and Surg. Journ., Jan. 1857.

§ Ibid., Jan. 1855, p. 48.

|| Boston Med. and Surg. Journ., April, 1855.

¶ Stethoscope, Feb. and March, 1854.

*W. J. Burnett, M.D.*—"Remarks upon the Presence of some Infusoria in the Tissues and Secretions of Patients Dying of Cholera;" Am. Journ. of Med. Science, Jan. 1850.

*J. H. Vanderveer.*—"Epidemic Cholera as it Appeared at the Franklin Street Hospital during the Summer of 1854;" New York Journ. of Med., Sept. 1855.

A committee, consisting of Drs. Jackson, Neill, Henry H. Smith, and William Pepper, appointed by the Philadelphia College of Physicians, reported on the state of the intestinal mucous membrane, in persons dying of cholera, as follows:—

"In the recent subject, the peritoneal coat, like all the serous membranes, was in all remarkably dry. The lubricating serosity is deficient in the serous membranes.

"The epithelial layer of the mucous membrane was, in all the specimens, either entirely removed or was detached, adhering loosely as a pulpy layer mixed with mucus or an albuminoid substance.

"Peyer's glands were developed to a greater or less extent in all the cases examined.

"The solitary glands were also developed, and contained, in the recent subject, a minute quantity of white substance.

"The villi are denuded of the epithelial covering, but are unchanged in other respects.

"The capillary vessels are entire, and manifest no departure from their normal state."

Dr. Holmes* mentions that in one case of cholera, in which there was suppression of urine, he had discovered distinct crystals, having the exact forms of those of lithic acid; and Dr. W. Burnett, in the paper above quoted, describes the infusoria met with in persons dying of cholera; they were almost always present in the urine of nearly forty patients, but only rarely, as far as could be ascertained, in the tissues. In the seventy-eight autopsies spoken of by Dr. Vanderveer, the usual appearances found in cholera were noticed; in addition, it is stated that, in many instances the collection of serum in the ventricles was very great; in some, three ounces of serum were found in the ventricles alone, independent of what was collected between the pia mater and arachnoid membrane, and in the cranium.

---

### FEVERS.

*J. B. Upham.*—"Cases of Maculated Typhus or Ship Fever," (with plates;) New York Journal of Medicine, July, 1852.

*Austin Flint.*—"Clinical Reports on Continued Fever," pp. 126, 230, and 339, *et seq.*; Buffalo, 1852.

*R. La Roche.*—"Observations on the Black Vomit, its Nature and Composition, and its Value in the Diagnosis and Prognosis of Yellow Fever;" Am. Journ.

---

* Am. Journ. of Med. Science, July, 1850.

of Med. Science, January and April, 1854; and chapter on this and on Anatomical Lesions in his work on Yellow Fever.

*Alonzo Clark.*—"On Black Vomit, and on the state of the Liver in Yellow Fever;" New York Medical Times, May, 1853; also page 370 of fourth edition of Bartlett on the Fevers of the United States.

*Myddleton Michel.*—"Microscopic Researches on the Black Vomit of Yellow Fever;" Charleston Medical Journal and Review, May, 1853.

*Thomas Hewson Bache.*—"Observations on the Pathology of Cases of Yellow Fever admitted into the Pennsylvania Hospital during the summer of 1853;" Am. Journ. of Med. Science, July, 1854.

*Samuel A. Cartwright.*—"Post-mortem Examination in the Yellow Fever of Natchez;" New Orleans Med. and Surg. Journal, March and May, 1857.

*Alonzo Clark.*—"On the Liver in Remittent Fever;" in Bartlett, on the Fevers of the United States, fourth edition, p. 370, and in appendix to Dr. La Roche's work on Yellow Fever.

*N. S. Davis.*—"On the Changes which take place in the Blood in the Continued Forms of Fever;" Transactions of the Illinois State Medical Society for the year 1857.

*W. A. Hammond.*—"On the Alterations Induced by Intermittent Fever in the Physical and Chemical Qualities of the Urine, etc.;" Am. Journ. of Med. Science, April, 1858.

*Joseph Jones.*—"Observations on Malarial Fever;" Southern Med. and Surg. Journal for 1858.

*H. M. Stuart.*—"An Investigation of the Urine in Remittent and Intermittent Fevers, proving the Hyperphosphatic State of the Blood, also the Eliminating Properties of Quinine;" Charleston Medical Journal, May, 1857.

It will be seen from the number of authors quoted, that the morbid alterations in fevers have received a very considerable amount of attention. Some few vague and theorizing papers have appeared on the subject, but the majority are based on research and careful observation. To render the record more complete, we have introduced matter which, perhaps, more appropriately would have belonged to the different organs we have already examined.

*Continued Fever.*—In a paper on *typhus* fever, based on a series of observations made during the prevalance of this disease at South Boston and Deer Island Hospitals in 1847 and 1848, Dr. J. P. Upham has carefully depicted the morbid appearances found in the intestine. He describes especially a peculiar intestinal affection, which is to be regarded as secondary, and which commonly makes its appearance during the latter stages of convalescence. Peyer's glands remain unaffected, but the solitary glands are raised, inflamed, and sometimes ulcerated. In the small intestine, especially the ileum, well-marked thickened ridges are perceived; in the interspaces, between the ridges, the mucous membrane may be brilliantly injected or congested. In some cases the large intestine shows its mucous lining throughout injected and thickened. Even in the acute stage of the fever, congestion and discoloration of the intestines occur. In the chapters indicated of Dr. Flint's essay on fevers, the post-mortem results of cases of continued fever are described with the author's usual accuracy.

Dr. Davis, in the article above quoted, after referring to the analyses of Andral and Gavarret of the blood of typhoid fever patients, gives the following analyses of his own in a case in which the microscope showed the blood, soon after it was drawn, to contain red corpuscles, more variable in size and more irregular in appearance than in healthy blood: the proportion of the elements expressed in 1000 parts were—water, 812·42 parts; red corpuscles, 106·77; albumen, 67·27; fibrin, 2·75; fatty matter, 0·67; saline, 10·09; total, 999·97. In an examination of a marked case of typhus, the amount of water was 791·0 parts; red corpuscles, 130·1; albumen, 67·0; fibrin, 1·9; extractive and salts, 10·0. "Taking the average," the author says, "of twelve analyses of blood from as many different patients with continued fever, I found the relative proportion of the different constituents or proximate elements very nearly normal. Taking separate cases, I find the blood taken from such as have been accompanied by more than the ordinary amount of water of intestinal discharges, to exhibit an excessive proportion of red corpuscles, while the reverse is true of such as have little or no such excess of intestinal evacuations; hence every analysis, to be valuable, must be accompanied by a detailed history of the case. While we find comparatively little change in the amount or relative proportion of the several proximate elements of the blood in continued fever, the alterations in quality are striking and uniform. Thus, in every case examined, the color was darker and more purple than normal; the fibrin coagulated very slowly, contracted but little, and the fibrin formed by the coagulation possessed less tenacity than in healthy blood, while the red corpuscles, viewed under the microscope, appear less biconcave, less uniform in size, and exhibit less attraction or affinity for each other; and the white corpuscles appeared to be very deficient in number." It will be perceived from these remarks that the results obtained, especially with reference to the relative proportion of fibrin, (which was found very little below the normal standard,) differ from the statements of Andral. The process by which the fibrin was separated consisted in allowing the blood to remain at rest nearly twenty-four hours, thereby giving time for the clot to be fully formed. This was inclosed in a piece of fine linen cloth, and washed with water until nothing but the pure white fibrin was left on the cloth; it was then thoroughly dried and weighed.

Analyses of blood taken from patients laboring under attacks of simple *intermittent* and *remittent* fever, led to the conclusion that the qualities or properties of the several constituents of the blood are normal, but that the relative quantity of the different proximate elements of the blood undergo important changes. The red corpuscles are markedly, and nearly uniformly diminished in proportion to the duration of the disease; the white corpuscles also were deficient; the albumen and fibrin only slightly below the normal average. Here also the results differ from those obtained by MM. Andral and Gavarret.

Dr. Joseph Jones is at present contributing a series of extended and valuable observations on the blood, and the characters of the fluids in malarial fevers, which, as they are not yet concluded, we reserve for a further notice; in the mean time we direct attention to them. Dr. Hammond's analyses of the urine of intermittent fever have led him to the conclusion that, during the fever, the uric acid and the phosphoric acid are very much increased in amount, and the urea and chlorine greatly diminished. During the intermission, there is a close approach to the normal constituents of the urine, but a subsequent paroxysm restores the former condition; the sulphate of quinia, however, reverses it.

Dr. Stuart finds in the urine of intermittent fever a sediment consisting of uric acid, urate of soda, biliary matter, and mucus. After quinia had been taken, all the above were increased in quantity, and the triple phosphates found in addition. The author believes that the blood is in a hyper-phosphatic state, and that quinia acts as a renal depurant.

*Remittent Fever.*—The limits we have set ourselves prevent us from going farther back than the year 1850, or we should have had to analyze the valuable papers of Drs. Stewardson,* Swett,† and Stillé‡ on the morbid appearances witnessed in this fever. Dr. Clark has found the cause of the peculiar color in the bronze or olive liver of remittent fever, to depend upon an infinite number of colored microscopical particles of irregular shape and size, varying in hue from red or orange to an opake jet black; some are beautifully crystallized, while some are in globular dots and grains; the black, often in globular grains, may also be seen in friable semi-crystalline scales, some of them almost large enough to be seen by the naked eye. The nature of this coloring matter must be sought among the chemical transformations of the hæmatin or coloring matter of the blood. They may be classed together under the group of pigment matter, to which the term hæmatoidin has been given. Specimens kept in alcohol and water lose, but slowly, something of their peculiar hue; especially is this the case if the color depends on the crystalline and red varieties.

*Yellow Fever.*—Dr. La Roche has in two articles described the physical characters of *black vomit*, and has brought together the views entertained as to its production. He also depicts the microscopic characters of black vomit from specimens examined by Prof. Leidy. Broken-down blood corpuscles and epithelial cells, varying in size and shape, were met with in all. A chemical examination of the vomit by Prof. Rogers, yielded albumen, sulphuric acid in a state of combination, chlorine, alkaline bases, earthy phosphates, iron, and hydrochloric acid in the free state. Dr. Myddleton Michel concludes from the examination of black vomit he has made, "that it is blood chemically altered by the action of acids, whether in or out of the vessels, the acids being hydrochloric when derived from the stomach, sulphuric acid§ of the sulphuretted hydrogen,(?) or carbonic and lactic acid, when obtained throughout the remaining portions." The pasty stools are composed of the black matters which have lost all of their characteristic features but the color, having undergone a process of digestion. No animalculæ are describable in either fresh or putrescent black vomit, but as it decomposes, certain fungi are disclosed, the result of fermen-

---

* Am. Journ. of Med. Science, April, 1841.

† Ibid., January, 1845. ‡ Ibid., April, 1846.

§ This must be a misprint, to which we would call the author's attention.

tation. The author further states that the artificial black matter produced by the addition of an acid to blood, is in every respect analogous to true black vomit, being both physically, chemically, and microscopically identical.

The absence of animalculæ is confirmed by Dr. Riddell,* who has, however, met with a form of sarcinæ in the ejected fluid. Dr. Clark has noticed, in specimens of black vomit, blood corpuscles of a uniform dark yellowish-brown color, some in the process of disintegration, forming nebular spots of considerable size; numerous scales of opake black matter, having no uniform shape, susceptible of fracture in any direction, and probably the product of some chemical change in the coloring matter of the blood; a quantity of a peculiar crystalline matter, sometimes in the form of a brush, but oftener flattened, white in color, and nearly transparent; and lastly, a vegetable growth in linear, and rather stout joints, the sections commonly separated, but often united as by a hinge. This element disappeared entirely after the lapse of three weeks.

A still more valuable observation than the preceding is the one by the same author on the liver of yellow fever. Since the time of Louis's researches, no contribution has been made to the subject which in importance or interest compares with this, and which reflects more credit on the observer. Dr. Clark has demonstrated that the cause of the yellow color of the liver in yellow fever is owing to a fatty state of the organ, probably an acute fatty degeneration. Dr. La Roche, in the most complete of all the monographs published on yellow fever, mentions that Dr. Leidy had confirmed Dr. Clark's statement in nine cases. Very important as bearing on this point, are also the autopsies of Dr. T. H. Bache, made at the Pennsylvania Hospital. In his admirable paper on yellow fever, he gives a summary of the post-mortem appearances of fourteen cases of yellow fever: in all, the liver, when examined with the microscope, was found in a state of fatty change. Generally, the cells were so studded with oil globules, as to give the idea of looking at a number of these latter, which had by chance become agglomerated or entangled by granular matter. Nor did the oil globules confine themselves merely to the granular part of the cells and their nuclei, but they were found, of various sizes, floating freely all over the field of the microscope. Dr. Riddell, on the other hand, states that well-marked fatty degeneration occasionally appears, but not constantly. This, however, does not invalidate Dr. Clark's opinion that the cause of the yellow color in the yellow fever liver is an extraordinary and rapid deposit of oil in the cells and tissues. Analogy, Dr. Clark believes, suggests that the main exciting cause of the oily deposit is an hyperæmic state of the organ during the fever. Dr. Joseph Jones,† in a letter to Prof. Samuel Jackson, states that in the liver of yellow fever patients he has found a substance which gave reactions similar to cellulose, and which presented, under the microscope, an appearance like the granules of starch.

Dr. Cartwright has placed on record post-mortem examinations made during the yellow fever of Natchez. In some of the subjects intussusception of the bowels, probably cadaveric, is noted. The bodies were examined immediately after the extinction of life. Morbid appearances were observed in the ganglionic system, and, in some cases, in the supra-renal capsules.

---

* New Orleans Medical and Surgical Journal, November, 1852.

† Am. Journ. of Med. Science, October, 1857.

